# Correction: Quantitative Analysis of Respiration-Related Movement for Abdominal Artery in Multiphase Hepatic CT

**DOI:** 10.1371/journal.pone.0131794

**Published:** 2015-06-25

**Authors:** The PLOS ONE Staff

## Notice of Republication

This article was republished on June 9, 2015, to replace an incorrectly published version of the manuscript. In addition to textual changes, the correct version includes author Shing-Hong Liu. Please download this article again to view the correct version. The originally published, uncorrected article and the republished, corrected article are provided here for reference.

## Supporting Information

S1 File Originally published, uncorrected article. (PDF)

S2 File Republished, corrected article. (PDF)

## References

[1] Lin Y-H, Huang S-M, Huang C-Y, Tu Y-N, Liu S-H, Huang T-C (2014) Quantitative Analysis of Respiration-Related Movement for Abdominal Artery in Multiphase Hepatic CT. PLoS ONE 9(12): e114222 doi: 10.1371/journal.pone.0114222 25536144 10.1371/journal.pone.0114222 PMC4275208

