# Supplementary material for: Correction: Quantitative Analysis of Respiration-Related Movement for Abdominal Artery in Multiphase Hepatic CT
Source: PLoS One. 2015 Jun 25;10(6):e0131794. doi: 10.1371/journal.pone.0131794 (PMC4482259; doi:10.1371/journal.pone.0131794)
Supplement: S2 File — (PDF) [file pone.0131794.s002.pdf]

RESEARCH ARTICLE

Quantitative Analysis of Respiration-Related Movement for Abdominal Artery in Multiphase Hepatic CT

Yang-Hsien Lin[1], Shih-Min Huang[2], Chin-Yi Huang[3], Yun-Niang Tu[3], Shing-Hong Liu[4], Tzung-Chi Huang[1,5]*

1 Department of Biomedical Imaging and Radiological Science, China Medical University, Taichung City, Taiwan, **2** Department of Radiology, China Medical University Hospital, Taichung City, Taiwan, **3** Department of Diagnostic Radiology, Peng Hu Hospital, Ministry of Health and Welfare, Peng Hu City, Taiwan, **4** Department of Computer Science and Information Engineering, Chaoyang University of Technology, Taichung, Taiwan, **5** Department of Biomedical Informatics, Asia University, Taichung City, Taiwan

* tzungchi.huang@mail.cmu.edu.tw

OPEN ACCESS

Citation: Lin Y-H, Huang S-M, Huang C-Y, Tu Y-N, Liu S-H, Huang T-C (2014) Quantitative Analysis of Respiration-Related Movement for Abdominal Artery in Multiphase Hepatic CT. PLoS ONE 9(12): e114222. doi:10.1371/journal.pone.0114222

Academic Editor: Qinghui Zhang, University of Nebraska Medical Center, UNITED STATES

Received: September 9, 2014

Accepted: November 4, 2014

Published: December 23, 2014

Copyright: © 2014 Lin et al. This is an open access article distributed under the terms of the Creative Commons Attribution License, which permits unrestricted use, distribution, and reproduction in any medium, provided the original author and source are credited.

Data Availability Statement: All relevant data are within the paper and its Supporting Information files.

Funding: This study was financially supported by the school project of China Medical University, Taiwan, and by Taiwan Ministry of Health and Welfare Clinical Trial and Research Center of Excellence (DOH102-TD-B-111-004). The funders had no role in study design, data collection and analysis, decision to publish, or preparation of the manuscript.

Competing Interests: The authors have declared that no competing interests exist.

Abstract

Objectives

Respiration-induced motion in the liver causes potential errors on the measurement of contrast medium in abdominal artery from multiphase hepatic CT scans. In this study, we investigated the use of hepatic CT images to quantitatively estimate the abdominal artery motion due to respiration by optical flow method.

Materials and Methods

A total of 132 consecutive patients were included in our patient cohort. We apply the optical flow method to compute the motion of the abdominal artery due to respiration.

Results

The minimum and maximum displacements of the abdominal artery motion were 0.02 and 30.87 mm by manual delineation, 0.03 and 40.75 mm calculated by optical flow method, respectively. Both high consistency and correlation between the present method and the physicians' manual delineations were acquired with the regression equation of movement, $y = 0.81x+0.25$, $r = 0.95$, $p<0.001$.

Conclusion

We estimated the motion of abdominal artery due to respiration using the optical flow method in multiphase hepatic CT scans and the motion estimations were validated with the visualization of physicians. The quantitative analysis of respiration-related movement of abdominal artery could be used for motion correction in the measurement of contrast medium passing though abdominal artery in multiphase CT liver scans.

Introduction

Multiphase dynamic computed tomography (MDCT) imaging with intravenous injection of contrast medium is essential for the diagnosis of hepatocellular carcinoma (HCC) and in the follow-up examination for patients undergoing treatment of liver tumors [1]. Multiphase hepatic CT scan using intravenous infusion of a contrast medium for the examination of the entire liver during unenhanced phase, arterial phase, portal venous phase and delayed phase has been proven to improve the evaluation of the hemodynamics of hepatic tumors and for the differential diagnosis of hepatic tumors [2–3]. Clinically, the automated bolus-tracking programs (e.g. Smart Prep) monitoring the intervenous contrast enhancement detects the contrast medium moving in abdominal artery on multiphase CT liver scans [4]. Meanwhile, the respiration-induced motion in the liver causes potential errors on the measurement of contrast medium in abdominal artery from multiphase hepatic CT scans [5–6]. The inaccurate Hounsfield unit measurement in moving abdominal artery resulted from respiration was shown in Fig 1 as an example.

The respiratory motion of the upper abdominal arteries in vascular interventions was investigated and reported in several studies [7–8]. Strategies to compensate for breathing motion include the use of 4D imaging techniques, gating technique and liver immobilization. The

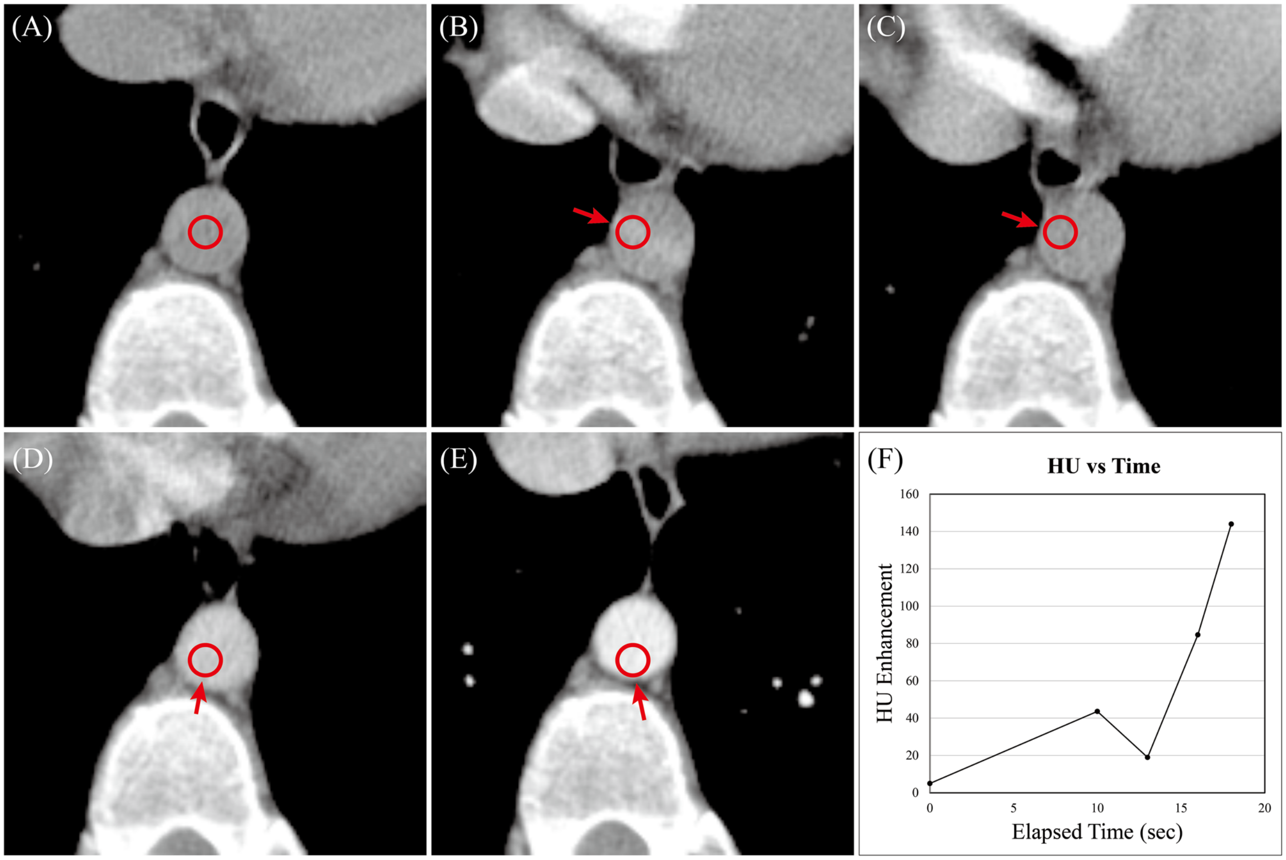

Fig 1. An example to show the clinical challenge on flow measurements in the abdominal artery with respiratory motion. (A) the ROI of abdominal artery at baseline phase of multiphase hepatic CT (B) the first scan of the monitor phase CT with ROI drawn at the baseline phase. (C)(D)(E) the consecutive CT scans in monitor phase showing the abdominal artery moving around with the inaccurate location (red arrows) while using the static ROI. (F) the flow measurement of contrast medium through the artery, Which illustrates the misalignment of abdominal artery during the bolus tracking process. A series of CT scan images dynamically detect the contrast medium through the artery at the same single slice, but the respiratory motion causes the inaccurate locations by ROIs (red arrows).

doi:10.1371/journal.pone.0114222.g001

deformable registration to calculate the trajectories of abdominal organs using 4D CT for motion estimation has been described by Hallman *et al* [9]. The gating techniques using real-time position management and active breathing control to limit the uncertainty contributed by respiration for PET imaging and radiotherapy were reported in previous studies [10–14]. These studies successfully utilized the temporal information to resolve the respiratory effects in imaging. However, studies investigating the quantification of respiratory motion and its influences on dynamic blood flow measurement using multiphase hepatic CT scan is still absent. A multiphase hepatic CT data set consists of consecutive images with contrast medium moving in abdominal artery which possesses complete temporal information. In the present study, we used hepatic CT images to quantitatively estimate the abdominal artery motion due to respiration by deformable image registration. The motion estimations were evaluated and compared by the physician's visualization. The quantitative motion estimation can be applied in motion correction for clinical diagnosis of HCC using MDCT.

Materials and Methods

Patient population

In this retrospective study, we included patients who underwent contrast-enhanced liver MDCT for the initial diagnosis or follow-up of confirmed HCC between July 2013 and December 2013. A total of 132 consecutive patients (84 men and 52 women; age range, 35–88; mean, 63 years) were identified and selected (Table 1). All patient identifiers were removed from the images for the study. This study was approved by the Institutional Review Board of China Medical University, Taiwan, and informed consent was waived due to the retrospective nature of the study.

MDCT Protocols

The 132 selected cases underwent contrast-enhanced abdominal helical 64-MDCT examination using Smart Prep (GE Healthcare Lightspeed VCT, Waukesha, Wisconsin), which included 639 axial CT images at abdominal artery. The region of interest (ROI) cursor for Smart Prep was placed in the abdominal artery and the threshold of ROI was set to 130 HU for the starting scan phase.

The protocols of the baseline and monitor phases were operated at 120 kV tube voltage, 40 mA tube current and 0.6 second rotation time to reconstruct the CT images with 5 mm slice thickness and 0.82 mm/pixel spatial resolution. Monitoring delay time and inter scan time were set to 10 and 3 second respectively in the monitor phase. The iodinated contrast media was administrated at the rate of 3–3.5 (ml/s) with an automatic power injector (Optivantage DH; Mallinckrodt Imaging Solutions, Hazelwood, Mo) in all patients. The volume of contrast

Table 1. Characteristics of Population (N = 132).

Men	84
Age (yrs)	63±12
Weight (kg)	59±17
Cardiac Disease	36 (27)
Liver Cirrhosis	87 (66)
Suspected HCC	62 (47)
Known HCC	70 (53)

Note—all the data were presented as mean ± SD or n (%).
Abbreviation—HCC = hepatocellular carcinoma.

doi:10.1371/journal.pone.0114222.t001

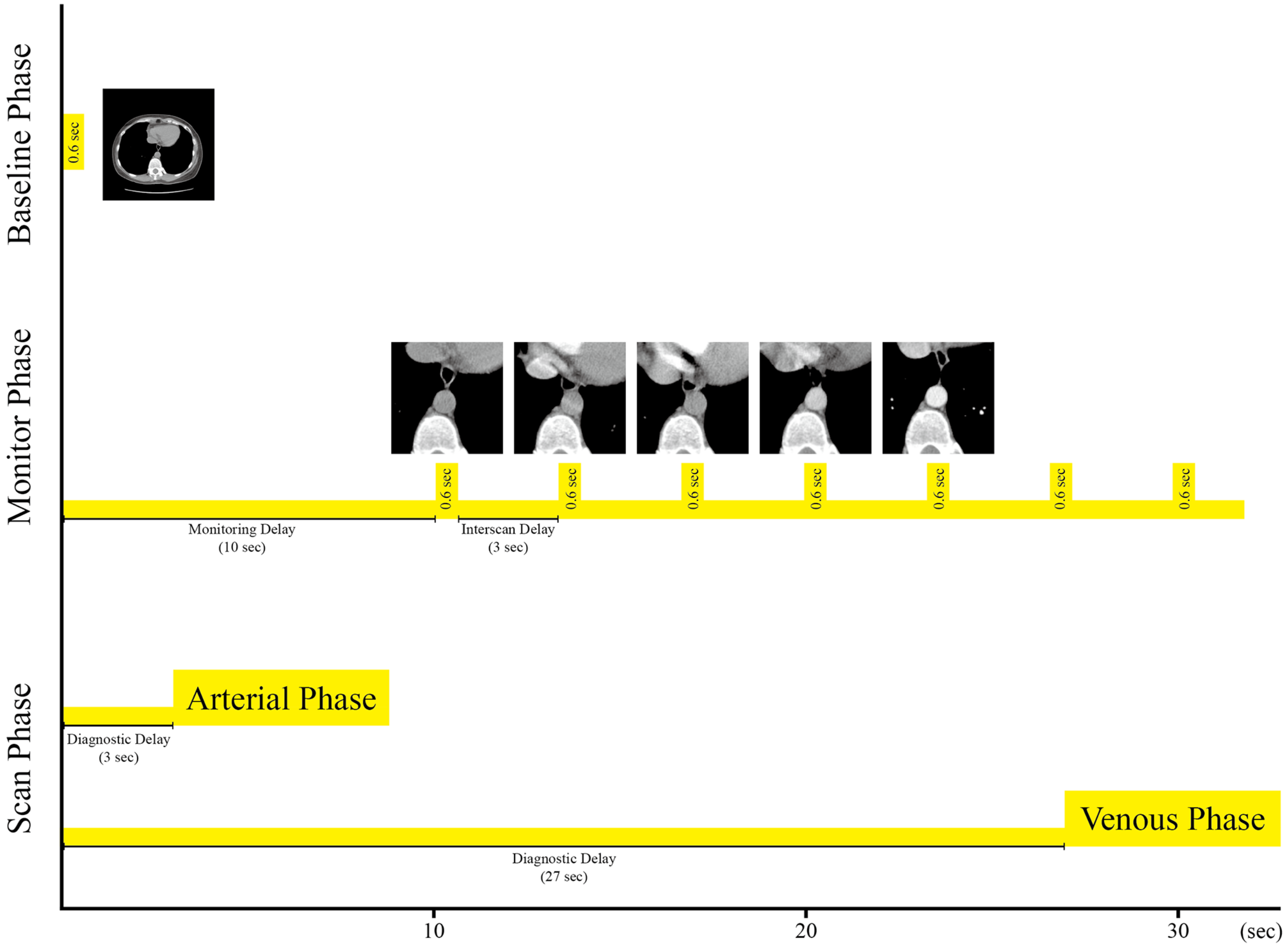

Fig 2. Multiphase CT scan protocol consists baseline, monitor and scan phases. A single slice was chosen in the baseline phase to place ROI, and the mean intensity of the ROI was measured in the monitor phase while IV contrast was injected. Scan phase was triggered when enhancement reached the threshold value.

doi:10.1371/journal.pone.0114222.g002

agent was calculated based on the patient's body weight, with the total range from 80 to 120 mL. Multiphase CT scan protocol is presented in Fig 2.

Definition of the Barycenter of Vessel Section

The data sets were randomized and interpreted independently by two radiologists with 12 (YN Tu) and 15 (CY Huang) years of post-training experience at interpreting CT images and delineating the contour of the abdominal artery. The scans displayed at standard window settings (width, 350HU; level, 40 HU) for definition the location of the artery. Manual delineation was performed on Image J (http://rsb.info.nih.gov/ij/) and the barycentric coordinates C of the artery were calculated as following:

$$C(a,b) = \left(\frac{\sum_{1}^{n} x_i}{n}, \frac{\sum_{1}^{n} y_i}{n} \right)$$

, where C denotes the barycenter in the section, n is the total pixel number in the ROI, and (x_i, y_i) was the coordinates of the ith pixel in the ROI.

Therefore, the direction and displacement of the abdominal artery was calculated from successive barycenter, the equation is presented as:

$$\Delta v = C_2 - C_1 = (a_2 - a_1, b_2 - b_1)$$

, where C_1 and C_2 are the barycenter from successive cross-section image, and $(a_2\text{-}a_1, b_2\text{-}b_1)$ is the change of barycenter in horizontal and ventricle axis.

Estimation of Motion Vector Field

Optical flow method (OFM), an image intensity gradient based method that is capable to accurately calculate the motion vector field (MVF) from an image sequence, estimates the local motion based upon local derivatives in the sequence of images. We apply the 2-dimensional optical flow algorithm attributed to Horn and Schunck [15] to compute the MVF of the abdominal artery which includes anterior-posterior (AP) and lateral (LAT) displacements for each pixel in CT images. This algorithm combined the gradient constraint with a global smoothness term to constrain the estimated velocity field. The iterative equation, shown as the following, was used to calculate the MVF:

$$v^{(n+1)} = v^{(n)} + \nabla f \left(\frac{\nabla f \cdot v^{(n)} + \frac{\partial f}{\partial t}}{\alpha^2 + \|\nabla f\|^2} \right)$$

, where n denotes the iteration number, which was 100 typically in all estimations, $v(n)$ is the average displacement derived from the surrounding pixels, f is the image intensity, and α is the weighting factor, which is empirically set at 5 for the OFM [16].

In this study, v represents the movement velocity (mm/sec) based on contrast changes with interscan time for the anterior-posterior (AP) and lateral (LAT) directions. The total movement for each voxel within the abdominal artery is defined as $\sqrt{AP^2 + LAT^2}$. The OFM for motion estimation was validated in previous studies that compared the OFM measurements with simulations and visual inspection [17–20].

Statistical Analysis

Quantitative analysis of the displacement of the abdominal artery was based on manual delineation and optical flow method on the CT monitoring phase. First, correlation of measurements by the two observers was determined with the Pearson correlation coefficient. Furthermore, the agreement and consistency of two methods were evaluated in this study. In the statistical analysis, the MedCalc software (MedCalc Software, Mariakerke, Belgium) was used to compare the results between the manual delineation and OFM. Passing-Bablock regression and Bland-Altman plot were used to evaluate the correlation and agreement between the manual and automatic estimations.

Radiation Dose

Rotation time and tube current were set 0.6 second and 40 mA for image acquisition. Radiation exposure dose is 24 mAs with a $CTDI_{vol}$ of 2.81 mGy for each slice on patients. The net mean number of scans for helical CT was 5 for each patient. Mean $CTDI_{vol}$ was 14.05 mGy based on SmartPrep.

Table 2. Motion estimation of the abdominal artery.

		Average Displacement[a] (mm)	
Radiologist	Area (mm^2)[b]	AP[b]	LAT[b]
1	597±235 (259–1498)	0.16±2.69 (-28.78–7.40)	0.23±2.26 (-13.27–16.10)
2	600±238 (233–1559)	0.16±2.78 (-28.87–7.94)	0.21±2.30 (-12.76–16.35)

Note—all the displacement data were presented as mean ± SD.

Abbreviation—AP = anterior to posterior view, LAT = lateral view.

[a]Derived from two radiologists.

[b]*p*-value<0.05.

doi:10.1371/journal.pone.0114222.t002

Results

Motion estimation of the abdominal artery with the ROIs was shown in Table 2. No significant differences were observed in both AP and LAT displacements between the two radiologists. In this study, the minimum and maximum displacement of the abdominal artery motion were 0.02 and 30.87 mm by manual delineation, 0.03 and 40.75 mm calculated by OFM, respectively (Table 3). The visualizations of the respiratory motion and MVF measurements in color scale for a selected patient on the axial view are presented in Fig 3. The correlation between the OFM and the physicians' manual delineations is shown in Fig 4A with the regression equation of movement, $y = 0.81x+0.25$, $r = 0.95$, $p<0.001$ and the Bland-Altman plot for OFM and the physicians' manual delineations is illustrated in Fig 4B. Both high consistency and correlation between the two methods were acquired.

Discussions

Motion studies and their synchronization with hepatic artery motion provide insights into interpretation of HCC diagnosis from multiphase hepatic CT. Respiratory motion during data acquisition in CT inevitably leads to artefacts which jeopardize the accuracy in the bolus tracking in the abdominal CT where vessel shifts and deforms during the respiratory cycle. Shallow breathing has also been proposed to be preferable for longer CT perfusion protocols in order to minimize breathing artefacts for patients who have difficulties holding their breath, especially in liver perfusion studies where tissue texture renders image registration challenging [21]. Smart Prep is an essential bolus tracking technique to visually monitor the contrast enhance level for technologists or radiologists. Despite Smart Prep ameliorate the limitation of conventional CT imaging, numerous factors would affect the quality of enhanced hepatic CT images. Respiratory problem is not only a problem in diagnostic radiology but also a conspicuous issue in radiological therapy. In clinical practice, the ROI is necessary to be placed on the detected

Table 3. Comparison of abdominal artery motion parameters.

	AP*	LAT*	Movement*
OFM	0.05±3.28 (-39.49–35.07)	0.16±2.54 (-15.60–21.05)	1.98±3.46 (0.03–40.75)
MD	0.06±2.60 (-28.06–26.09)	0.15±2.22 (-13.81–16.85)	1.86±2.88 (0.02–30.87)

Note—all the data were presented as mean ± SD and its range (mm).

**p*-value<0.001.

Abbreviation—AP = anterior to posterior view, LAT = lateral view, OFM = Optical flow method, MD = Manual delineate by physician.

doi:10.1371/journal.pone.0114222.t003

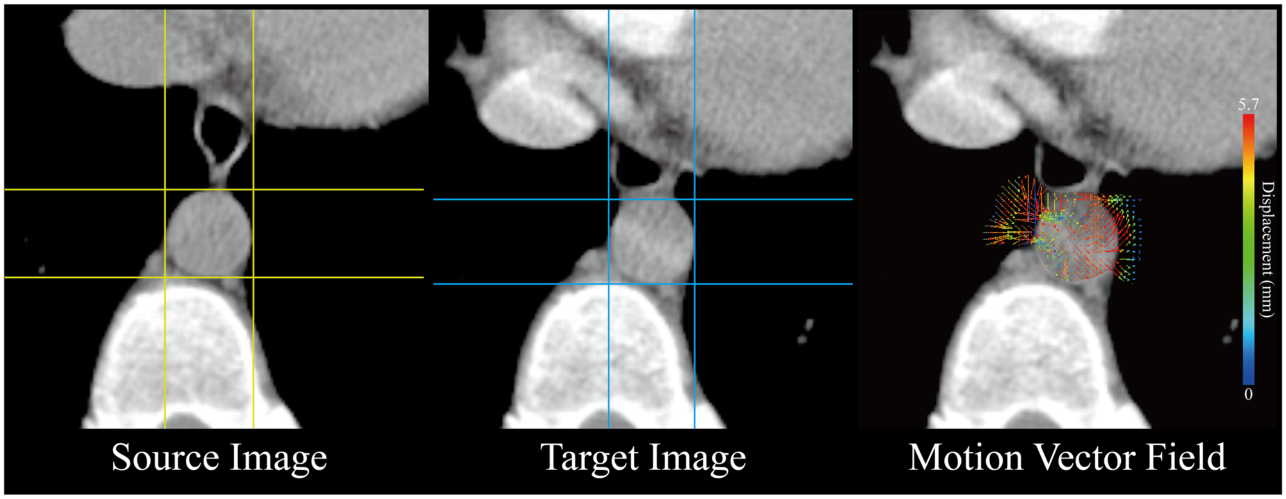

Fig 3. An example of the optical flow method estimation of motion vector field in abdominal artery on the axial view. (A) the source image (B) the target image taken 3 seconds after the source image. (C) the respiratory motion estimation presented by the motion vector field overlaid on the target image. The red and yellow colors indicate the high movement in abdominal artery.

doi:10.1371/journal.pone.0114222.g003

vessel as small as possible to avoid the interference caused by respiratory motion at monitoring phase. However, the flow information is limited by the small ROI which is only part of abdominal artery. In this study, an automatic method of quantitative respiration estimation using OFM is successfully validated by physicians' visualization. With quantitative respiration, the motion correction for multiphase hepatic CT scan is achievable, which improves the accuracy of contrast measurements and acquires the contrast enhancement in the dynamic hepatic CT images. To our best knowledge, the present study is among the first to report relative quantifications of respiratory motion of the hepatic artery using multiphase hepatic CT. The quantitative results provided the average movement of ROI was 1.98±3.46 mm, which was considered small motion. However, the maximum movement was 40.75 mm (Table 3). Even though a very small ROI was placed on the detected vessel to avoid the interference caused by respiratory motion at monitor phase, the large ROI motion causes that the incorrect location for

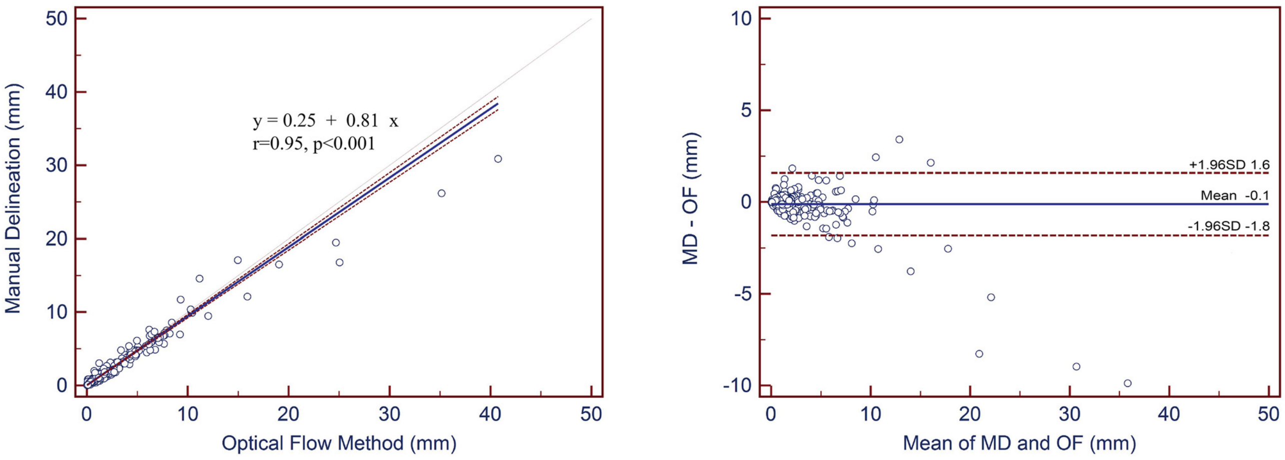

Fig 4. Passing-Bablock diagram and Bland-Altman plot to show the regression and consistency based on manual delineation (MD) and optical flow method (OF) results. OF demonstrates high correlation with the MD delineation results which were considered the standard.

doi:10.1371/journal.pone.0114222.g004

abdominal artery was placed in the corresponding ROI in other phases and the measurement of contrast information is inaccurate. Therefore, we still suggest that the motion correction of abdominal artery during the multiphase hepatic CT scan is needed to provide accurate objective diagnostic sign of hepatic tumors.

Free breathing makes the organ shift on CT scans. Respiratory motion makes the vessel moves dramatically on AP direction and mildly on LAT direction, relatively (Table 2 and Table 3). With calcification aorta, the ROI measurement in the abdominal artery occasionally fails due to respiratory motion. Currently, there is no general consensus amongst radiologists as to the optimal bolus tracking technique for multiphase hepatic CT examinations. Both methods—constant shallow breathing and multiple breath-hold sequences—have been utilized in previously published trials [22–24]. As breath-hold in CT scan is impossible on uncontrollable patients, respiratory correction via optical flow is a new insight to improve ROI missing in bolus tracking.

Manual delineation of abdominal artery was independently performed by two radiologists based on standard window (window level: 40; window width: 350) CT images. Pearson correlations illustrated the statistically insignificance of delineated area ($p<0.05$) and artery displacement ($p<0.05$). Thus, the manual delineation would not affect the accuracy in this study.

The difference between the maximum displacement of manual (30.87mm) and OFM (40.75 mm) raises the concern in the present study. Multiphase hepatic CT scan for the examination of the entire liver, including unenhanced phase, arterial phase, portal venous phase and delayed phase, was considered a long image acquisition. Patient movement during the imaging scan is another clinical issue besides the respiration. In present study, the motions due to respiration and patient movements were taken into account in motion estimation calculated by OFM. The OFM displacement is derived from the distribution of the apparent velocities of movement in the brightness patterns between the images. The dispersion effects from the patient movement may lead to errors in the estimation when the different motion models are included. In addition, the noise caused from beam-hardening artifact is included in low dose image scanning, especially MDCT imaging [25]. The quality of image is important while applying the optical flow method for motion estimation. Therefore, these influences could affect the uncertainty of estimation by optical flow method.

Conclusion

We estimated the motion of abdominal artery due to respiration using the optical flow method in multiphase hepatic CT scans and the motion estimations were validated with the visualization of physicians. Our results could be used for motion correction in the measurement of contrast medium passing though abdominal artery in multiphase CT liver scans. The correct hemodynamic information of in abdominal artery may facilitate the clinical diagnosis of HCC.

Author Contributions

Conceived and designed the experiments: YL. Performed the experiments: YL SH CH YT. Analyzed the data: YL TH. Contributed reagents/materials/analysis tools: YL SH TH SL. Wrote the paper: YL TH SL.

References

1. Merine D, Takayasu K, Wakao F (1990) Detection of hepatocellular carcinoma: comparison of CT during arterial portography with CT after intraarterial injection of iodized oil. Radiology 175:707–710. PMID: 2160675

2. Laghi A, Iannaccone R, Rossi P, Carbone I, Ferrari R, et al. (2003) Hepatocellular Carcinoma: Detection with Triple-Phase Multi—Detector Row Helical CT in Patients with Chronic Hepatitis 1. Radiology 226(2):543–549. PMID: 12563152
3. Yin MC (2012) Humans and cancer: An ongoing fight. BioMedicine 2(3): 83.
4. Kim T, Murakami T, Takahashi S, Tsuda K, Tomoda K, et al. (1999) Optimal phases of dynamic CT for detecting hepatocellular carcinoma: evaluation of unenhanced and triple-phase images. Abdom Imaging 24(5): 473–480. PMID: 10475931
5. Kubas A, Mornex F, Merle P, d'Hombres A, Lorchel F, et al. (2008) Irradiation of hepatocellular carcinoma: Impact of breathing on motions and variations of volume of the tumor, liver and upper abdominal organs. Cancer Radiother 12(8): 768–774. doi: 10.1016/j.canrad.2008.04.011 PMID: 18639479
6. Brandner ED, Wu A, Chen H, Heron D, Kalnicki S, et al. (2006) Abdominal organ motion measured using 4D CT. Int J Radiat Oncol Biol Phys 65(2): 554–560. PMID: 16690437
7. Clifford MA, Banovac F, Levy E, Cleary K. et al. (2002) Assessment of Hepatic Motion Secondary to Respiration for Computer Assisted Interventions. Computer Aided Surg 7:291–299. PMID: 12582982
8. Takemura A, Suzuki M, Sakamoto K, Kitada T, Iida H, et al. (2008) Analysis of respiration-related movement of upper abdominal arteries: preliminary measurement for the development of a respiratory motion compensation technique of roadmap navigation. Radiol Phys Technol 1:178–182. doi: 10.1007/s12194-008-0025-2 PMID: 20821144
9. Hallman JL, Mori S, Sharp GC, Lu HM, Hong TS, et al. (2012) A Four-Dimensional Computed Tomography Analysis of multiorgan Abdominal Motion. Int J Radiat Oncol Biol Phys 83(1): 435–441. doi: 10.1016/j.ijrobp.2011.06.1970 PMID: 22197238
10. Beddar AS, Kainz K, Briere TM, Tsunashima Y, Pan T, et al. (2007) Correlation between internal fiducial tumor motion and external marker motion for liver tumors imaged with 4D-CT. Int J Radiat Oncol Biol Phys 67(2): 630–638. PMID: 17236980
11. Wagman R, Yorke E, Ford E, Giraud P, Mageras G, et al. (2003) Respiratory gating for liver tumors: use in dose escalation. Int J Radiat Oncol Biol Phys 55(3): 659–668. PMID: 12573753
12. Liu F, Hu Y, Zhang Q, Kincaid R, Goodman KA, et al. (2012) Evaluation of deformable image registration and a motion model in CT images with limited features. Phys Med Biol 57(9):2539. doi: 10.1088/0031-9155/57/9/2539 PMID: 22491010
13. Zhang Q, Pevsner A, Hertanto A, Hu YC, Rosenzweig KE, et al. (2007) A patient-specific respiratory model of anatomical motion for radiation treatment planning. Med Physics 34(12):4772–4781. PMID: 18196805
14. Ford EC, Mageras GS, Yorke E, Ling CC (2002) Respiration-correlated spiral CT: a method of measuring respiratory-induced anatomic motion for radiation treatment planning. Medical physics 30(1): 88–97.
15. Horn BKP, Schunck BG (1981) Determining optical flow. Artif Intell 17:185–203.
16. Guerrero T, Zhang G, Huang TC, Lin KP (2004) Intrathoracic tumour motion estimation from CT imaging using the 3D optical flow method. Phys Med Biol 49:4147–4161 PMID: 15470929
17. Huang TC, Wang YC (2013) Deformation Effect on SUVmax Changes in Thoracic Tumors Using 4-D PET/CT Scan. PLoS One 8(3) e58886 doi: 10.1371/journal.pone.0058886 PMID: 23516568
18. Huang TC, Wang YC, Kao CH (2013) Thoracic Tumor Volume Delineation in 4D-PET/CT by Low Dose Interpolated CT for Attenuation Correction. PLoS One 8(9) e75903 doi: 10.1371/journal.pone.0075903 PMID: 24086662
19. Wu CC, Lin WC, Zhang G, Chang CW, Liu RS, et al. (2011) Accuracy evaluation of RBC velocity measurement in nail-fold capillaries. Microvasc Res 81(3)252–260. doi: 10.1016/j.mvr.2011.01.003 PMID: 21255589
20. Mok GS, Sun T, Huang TC, Vai MI (2013) Interpolated Average CT for Attenuation Correction in PET-A Simulation Study. IEEE Trans Biomed Eng 60(7) 1927–1934. doi: 10.1109/TBME.2013.2245132 PMID: 23392338
21. Kandel S, Meyer H, Hein P, Lembcke A, Rueckert JC, et al. (2012) Comparison of free breathing versus breath-hold in perfusion imaging using dynamic volume CT. Insights Imaging 3(4): 323–328. doi: 10.1007/s13244-012-0169-9 PMID: 22695948
22. Ippolito D, Sironi S, Pozzi M, Antolini L, Ratti L, et al. (2008) Hepatocellular carcinoma in cirrhotic liver disease: functional computed tomography with perfusion imaging in the assessment of tumor vascularization. Acad Radiol 15(7):919–927. doi: 10.1016/j.acra.2008.02.005 PMID: 18572129
23. Sahani DV, Holalkere NS, Mueller PR, Zhu AX (2007) Advanced hepatocellular carcinoma: CT perfusion of liver and tumor tissue—initial experience. Radiology 243(3):736–743. PMID: 17517931

24. Fleischmann D (2010) CT Angiography: Injection and Acquisition Technique. Radiol Clin North Am 48 (2): 237–247. doi: 10.1016/j.rcl.2010.02.002 PMID: 20609872

25. Utsunomiya D, Awai K, Tamura Y, Nishiharu T, Urata J, et al. (2006) 16-MDCT aortography with a low-dose contrast material protocol. American Journal of Roentgenology 186(2):374–378. PMID: 16423941
